# Histological and SEM Assessment of Blood Stasis in Kidney Blood Vessels after Repeated Intra-Arterial Application of Radiographic Contrast Media

**DOI:** 10.3390/life10090167

**Published:** 2020-08-27

**Authors:** Philipp Lamby, Alexander Minkow, Stefan Handt, Johannes Falter, Eva-Lotte Schellenberg, Stefanie Graf, Bernhard Hiebl, Silke Haerteis, Ole Gemeinhardt, Anne Krüger-Genge, Bernd Klosterhalfen, Ernst-Michael Jung, Ralf-Peter Franke, Arash Momeni, Lukas Prantl, Friedrich Jung

**Affiliations:** 1 Department of Plastic and Reconstructive Surgery, University Hospital Regensburg, 93053 Regensburg, Germany; johannes.falter@ukr.de (J.F.); stefanie1.graf@ukr.de (S.G.); lukas.prantl@ukr.de (L.P.); 2 Institute of Micro and Nanomaterials, University of Ulm, 89081 Ulm, Germany; alexander.minkow@uni-ulm.de; 3 Institute for Pathology, 52146 Bardenberg, Germany; handt@pathologie-aachen.de; 4 Department of Anesthesiology, University of Regensburg, 93053 Regensburg, Germany; eva-lotte.schellenberg@klinik.uni-regensburg.de; 5 Institute for Animal Hygiene, Animal Welfare and Farm Animal Behaviour, Virtual Center for Replacement–Complementary Methods to Animal Testing, University of Veterinary Medicine Hannover, 30173 Hannover, Germany; Bernhard.Hiebl@tiho-hannover.de; 6 Institute for Molecular and Cellular Anatomy, University of Regensburg, 93053 Regensburg, Germany; silke.haerteis@ur.de; 7 Charité-Universitätsmedizin Berlin, Humboldt-Universität zu Berlin, and Department of Radiology, Berlin Institute of Health, 10117 Berlin, Germany; ole.gemeinhardt@charite.de; 8 Department of Immunology, Faculty of Medicine, Dalhousie University, Halifax, NS B3H 4R2, Canada; anne.krueger-genge@iap.fraunhofer.de; 9 Institute of Pathology, 52351 Düren, Germany; bernd.klosterhalfen@web.de; 10 Department of Radiology, University Hospital Regensburg, 93053 Regensburg, Germany; Ernst-Michael.Jung@klinik.uni-regensburg.de; 11 Central Institute for Biomedical Engineering, Department of Biomaterials, University of Ulm, 89069 Ulm, Germany; rp.franke@web.de; 12 Division of Plastic and Reconstructive Surgery, Stanford University Medical Center, Stanford, CA 94304, USA; amomeni@stanford.edu; 13 Institute of Biotechnology, Brandenburgische Technische Universität Cottbus-Senftenberg, 01968 Cottbus, Germany; dihkf@saarmail.de

**Keywords:** acute kidney injury, nephrotoxicity, nephropathy, renal pathology, iodinated contrast media, electron microscopy, histopathology

## Abstract

Background: After application of iodinated contrast media (CM), a pronounced deterioration of the microcirculation in skin and myocardium was reported. Clinically, the repeated application of CM, especially, led to an increase of the renal resistance index (RRI). With respect to the transiency of the RRI increase, it is reasonable to assume that the deterioration of blood flow could be due to transient blood stasis caused by reversible morphologic cell alterations due to osmotic discrepancies between CM and human blood. Therefore, the hypothesis was investigated whether CM are able to induce in vivo such blood stasis and cell deformations in the renal vasculature of well-hydrated pigs. Methods: The in vivo study was performed as a prospective randomized examination to compare the effects of two different CM in 16 pigs (German Landrace). Pigs were randomized to receive either Iodixanol (*n* = 8), or Iopromide (*n* = 8). Each animal received 10 injections separated by 5-min intervals via the suprarenal aorta at a rate of 10 mL/s according to the usual procedure during a cardiac catheter examination. Finally, the kidneys were explanted and processed for histology (H & E staining and fibrin staining according to Weigert) as well as for scanning electron microscopy (SEM) with regards to morphologic correlates explaining the changes in the microcirculation. Results: In each of the predefined four categories of vascular diameters, blood stasis were found, but clearly more often after application of Iopromide than after application of Iodixanol (*p* < 0.001). In addition, Iopromide induced more blood stasis in all of the examined kidney regions compared to Iodixanol (*p* = 0.0001). There were no obstructive events in the middle cortex following the application of Iodixanol. Except for the region around a puncture channel of a placed-in catheter probe, no fibrin was detected in Weigert’s fibrin-stained samples, neither around the histologically assessed thrombi nor in vessels with blood stasis. Complementary SEM analyses revealed in a few cases only a slight generation of fibrin and thrombi and deformations, such as echinocyte and “box-like” deformations. Conclusions: According to previous in vitro studies, pathological erythrocyte deformations, such as echinocyte and box-like formation of erythrocytes, were observed also in vivo. In addition, blood stasis and/or thrombi could be detected in histological samples from explanted kidneys from young pigs after repeated in vivo administration of CM. In only a few cases, mural platelet aggregates within minimal fibrin meshes occurred only after the application of Iopromide.

## 1. Introduction

Contrast-induced acute kidney injury (CI-AKI) is associated with significant patient morbidity and mortality secondary to renal failure [1,2,3,4]. Hence, an improved understanding of CI-AKI is paramount if one is to reduce the incidence of these complications, including prolonged hospitalization, increased re-admission rates, and acute care dialysis [5,6,7,8,9,10,11]. In rare cases, persistent renal insufficiencies occur, resulting in chronical dialysis [12]. Vascular obstruction is discussed as one of the possible reasons [12].

Pre-existing renal injury is considered to contribute to the development of CI-AKI [8,13,14]. However, it has been demonstrated that the risk for CI-AKI among patients in the outpatient setting who repeatedly received contrast media (CM) [14] during coronary intervention [8] was similar to patients with chronic renal insufficiency.

An animal study in young and healthy pigs showed that repeated administration of CM can be associated with a transient increase in the “Renal Resistance Index” (RRI) [15]. This had been demonstrated before also in a clinical study [16]. The pig study revealed a transient stasis of the renal microcirculation in different kidney regions, which was more pronounced after administration of Iopromide vs. Iodixanol [17]. This was in line with another animal study in which could be shown that iodinated CM deteriorate the oxygen tension in the renal cortico-medullary region of pigs [18]. The studies [15,17,18] have been conducted within the same animal cohort of the presented study according to the 3R principle (refinement, reduction, and replacement).

A variety of manifestations in the microcirculation following CM administration has been reported. Several authors have demonstrated an impaired microcirculation in the skin following CM injection into the axillary artery in clinical studies [19,20,21]. A decrease of perfusion in porcine myocardium has, furthermore, been reported following CM injection into the left coronary artery [22]. Finally, a decrease of perfusion in the renal vasculature following repeated CM injections into the supra-renal aorta has been observed within in the same cohort of animals of the presented study [17]. As a possible reason for the CM-induced impairment of kidney perfusion, a constriction of the medullary vasa recta has been discussed [23], and this could be intensified by the formation of echinocytes [24,25,26,27,28,29,30] or by the buckling of endothelial cells [31].

Beyond the mechanisms reported above and particularly with respect to its transiency, the deterioration could be induced due to transient blood stasis in renal blood vessels. This potential mechanism, however, has not been examined to date.

Therefore, we investigated the kidneys from those animals that had been examined in vivo in the study reported by Lamby et al. [17]. Histological and complementary scanning electron microscopy (SEM) analyses were conducted to determine whether CM are able to induce stasis and/or the formation of thrombi in the renal vasculature following repeated supra-renal intra-aortal injections of two different CM.

## 2. Methods

### 2.1. Study Design

The in vivo study was performed as a prospective randomized examination to compare the effects of two different CM in pigs (*n* = 16). The details of animal housing and the experimental procedure of the study were published earlier [17]. Pigs were randomized to receive either Iodixanol (group I, *n* = 8) or Iopromide (group II, *n* = 8), and controls without CM were excluded by the local ethical board (AZ.: 54-2532.1-31/13).

To simulate the clinical routine, each animal received 16 g of iodine divided into 10 injections of 5 mL. Iodixanol 320 or 4.32 mL Iopromide 370 into the suprarenal aorta at 5 min intervals at a rate of 10 mL/s. Each animal received a total of 500 mL NaCl throughout the entire examination, which also reflects the clinical procedure.

The Bavarian Institutional Animal Care and Use Committee approved the study protocol (AZ.: 54-2532.1-31/13). All procedures were carried out in accordance with the guidelines and recommendations of the National Society for Laboratory Animal Science.

### 2.2. Animals

The young healthy German Landrace pigs were placed in supine position. Under sterile conditions, surgical access was gained through median laparotomy. After the intestine was displaced, the kidney was trans-peritoneally released from the surrounding fatty tissue and the fibrous capsule. The fibrous capsule had to be removed for measurement of the pO_2_ at the kidney surface with membrane sensors. Then, a catheter was inserted caudocranially into the abdominal aorta, with the tip lying 2–3 cm above the renal arteries for CM injection.

### 2.3. Iodinated Contrast Media

Two iodinated CM were applied: Iodixanol 320 mg Iodine/mL, GE Healthcare, Munich, Germany and Iopromide 370 mg Iodine/mL, Bayer/Vital, Berlin, Germany. Table 1 shows the characteristics of both contrast media.

### 2.4. Kidney Explantation

After the 10th CM injection and the last series of in vivo measurements (contrast enhanced ultrasound (CEUS) and oxygen tension (pO_2_), data published earlier [17,18]) both kidneys were explanted and marked with colored threads in a well-defined manner, so that the in vivo topographic kidney position was maintained throughout the subsequent procedures. The supplying vessels of 15 kidneys were not ligated, so that blood could flow freely out of the organs and only some blood remained in the lower parts of the kidneys next to the container bottom. The supplying vessels of one kidney were ligated, so that the blood remained in this organ. This allowed to visualize whether the cortical capillaries were filled with blood or not (this would not be possible in exsanguinated kidneys). Immediately after kidney explantation, the animals were euthanized by injection of potassium chloride.

Following explantation, radiographic investigations were obtained for an affiliated study [17] within a period of 30 min. Afterwards, the organs were immersed in 5 L of buffered formalin (4%). After exchange of the fixation solution, the organs were processed for histology. First, the kidneys were separated between the middle and the distal third of the kidney. From the ventral surfaces of the distal thirds of the kidneys, 5 mm-thick tissue blocks were excised with scalpels by experienced scientists who were not involved in the study (B.H. and O.G.); hence, both blinded to the objective of the study and, importantly, to which kidney had been exposed to which CM. They were asked to prepare samples allowing the analysis of the complete kidney tissue, including the cortex and medulla. Following the procedures described by Romeis [32], the tissue blocks were embedded in paraffin, from which 5 µm-thick tissue samples were prepared and either HE stained or fibrin stained according to Weigert’s method.

### 2.5. Hematoxylin and Eosin Staining (HE Staining)

#### 2.5.1. Microscopic Evaluation of The Samples

Stained samples from all of the right pig kidneys were completely evaluated in the cortex and medulla, moving methodically through the samples from the capsule in the direction of the papilla. A first set of 16 HE-stained samples was scanned in the microscope to assess the regional incidence of blood stasis in kidney blood vessels in the following regions: capsule-near cortex, the mid-cortex, the cortico-medullary transition, the mid-medulla, and the papilla-near medulla. In a second set of 16 samples, the diameters of vessels with blood stasis were quantified in these areas. Vessel diameters were measured using ImageJ (National Institutes of Health, Bethesda, MD, USA) [33]. In addition, the numbers of vessels with blood stasis were quantified. On average, 63 vessels were found in a 370 × 500 µm^2^ area, so that in one sample about 370 vessels could be evaluated.

#### 2.5.2. Staining Quality

**Figure 1:**
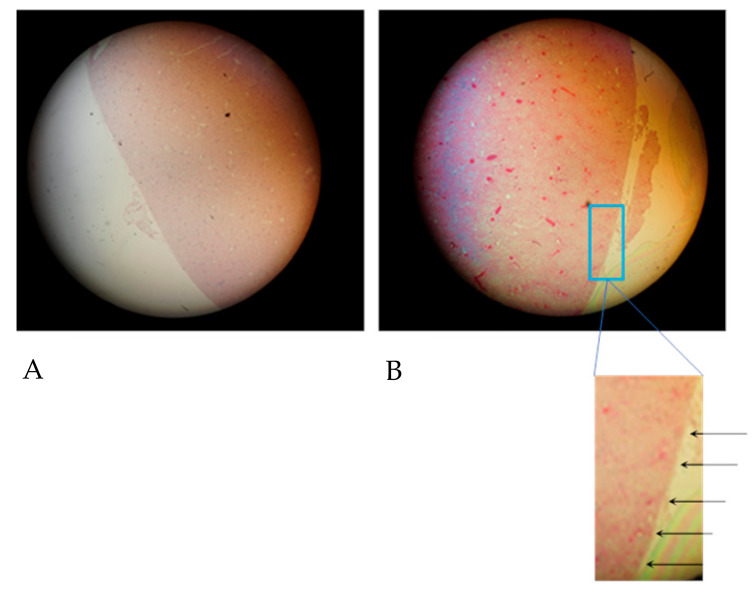
Hematoxylin and eosin (HE) stained kidney samples. (**A**) Blood-drained kidney, primary magnification 1:16. (**B**) Blood-filled kidney (supplying vessels ligated); the arrows in the insert are directed to blood-filled capillaries in the capsule of the kidney-primary magnification 1:16.

The quality of the hematoxylin-eosin staining (HE staining) according to Romeis [32] is demonstrated in Figure 1.

Figure 1A shows the cortical aspect of an HE-stained sample from a kidney after 10-fold injection of Iodixanol, when the supplying blood vessels were not ligated so that blood could flow freely from the vasculature. Cortical blood vessels filled with blood are shown in Figure 1B, when the supplying blood vessels had been ligated after the injection of 10 doses of Iodixanol.

The detail below Figure 1B shows that cortical capillaries were generally filled with blood almost up to the capsule (arrows).

### 2.6. Fibrin Stain According to Weigert

The histological fibrin staining according to Weigert was applied for the assessment of fibrin [33]. The effectivity of the Weigert fibrin staining was demonstrated in coagulated blood. Here, the fibrin appeared in gray-violet to brownish-violet colors.

Gray-bluish parts containing fibrin alone could be detected and red-brownish to yellow areas containing fibrin mixed with more or less erythrocytes.

Double refringence in polarized light illumination allowed the discrimination of s-like undulating bluish-violet fibrin fibers in gray-bluish colored areas covered with fibrin alone, and s-like undulating red-brown-violet fibrin fibers in red-brown-yellow areas covered with a mixture of fibrin and blood cells.

### 2.7. Detection of Blood Stasis

**Figure 2:**
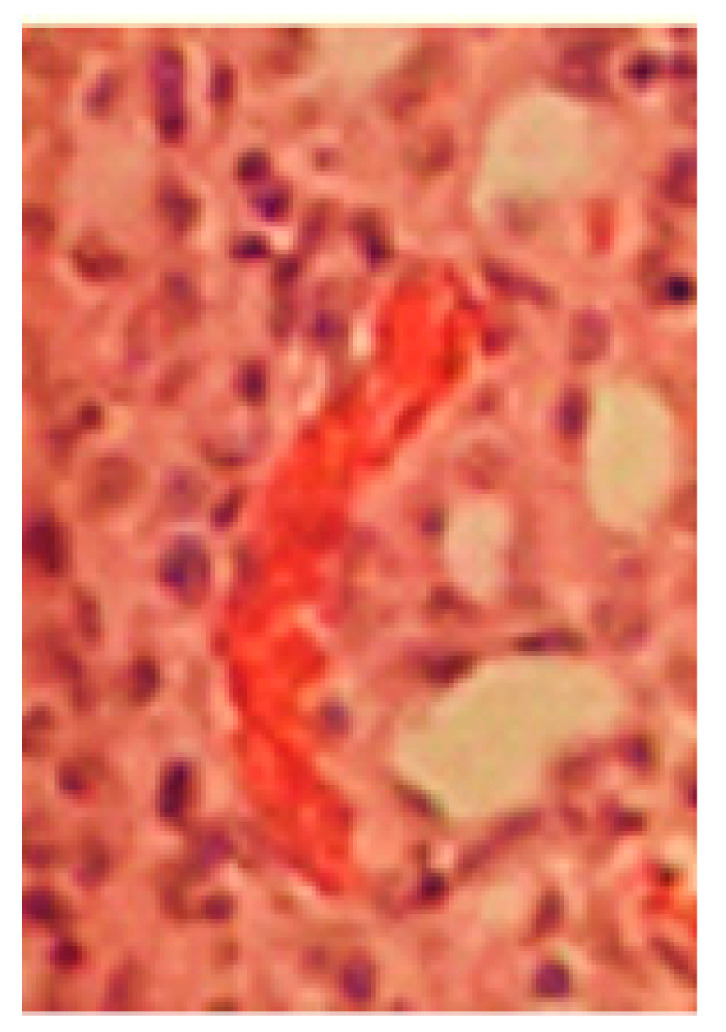
Details from a microphotograph in transillumination of blood stasis in a HE-stained tissue sample removed from the kidney medulla after application of Contrast Media. Primary magnification 1:200 (blood stasis with well recognizable singular integral erythrocytes in a congested blood vessel where erythrocytes appear in red color).

Blood stasis is recognized in the tissue samples presented in Figure 2 when only one or a few blood vessels were more or less filled with erythrocytes, and the majority of blood vessels was devoid of erythrocytes. Most importantly, erythrocytes had to be recognized as integral cells in cases of blood stasis.

### 2.8. Preparation of Samples for Scanning Electron Microscopy (SEM)

Two tissue blocks of each series of eight tissue blocks, from which the samples for HE- and fibrin staining had been extracted, were used to prepare the samples for SEM analyses. The 4 blocks were de-embedded out of paraffin, separated, and then re-embedded in paraffin. Using a 4 mm Ø trephine, three round tissue blocks were extracted out of each block from the regions “middle medulla”, “cortico-medullary transition”, and from the “subcapsular cortex”. The punched round samples were embedded separately in paraffin, and from these blocks, 1 µm-thick sections were extracted with a microtome, de-embedded out of paraffin, and mounted on ultra-pure silicon wafers. On samples and wafers an 8 nm-thick carbon layer was deposited (BalTec Med 020) and analyzed in the SEM system (LEO 1550, Fa. Carl Zeiss AG, Jena, Germany). This SEM system is in the production line of high-resolution Gemini analysis systems and equipped with a field-emission source and advanced sensor devices, allowing a resolution of 1.2 nm.

### 2.9. Statistics

Samples were described with the arithmetic mean and standard deviation. The null hypothesis was that there were no differences in the incidence of blood stasis in kidney blood vessels after the application of Iodixanol vs. Iopromide. To test this hypothesis, a variance analysis was performed; in the case of paired samples, an ANOVA for repeated measures (without Bonferroni adjustment, due to the explorative character of the study); in the case of unpaired samples, a one-factorial ANOVA. Probabilities less than *p* = 0.05 were considered as marked.

## 3. Results

### 3.1. HE Staining

#### 3.1.1. Blood Stasis in Kidney Vessels

The frequency of blood stasis in vessels of the 15 (ensanguined) explanted kidneys differed significantly for the two CM. After the 10 boli of Iodixanol, blood stasis was found in 62 vessels, while this was the case in 405 vessels after the application of Iopromide (*p* = 0.00485). The vessel diameter also proved to be a significant factor influencing the occurrence of stasis (ANOVA: *p* < 0.001). In capillaries, there were clearly less events than in vessels with diameters between 15 and 30 µm (*p* = 0.001); however, this was more than in larger vessels with diameters greater than 300 µm (*p* = 0.012). Most events of blood stasis occurred in vessels with diameters between 15 and 30 µm. This was notable in comparison to capillaries (*p* = 0.001) and to vessels with diameters larger than 300 µm (*p* = 0.0001). In vessels with diameters between 30 and 300 µm, more events occurred than in vessels with diameters larger than 300 µm (*p* < 0.0001). Table 2 shows the frequencies of blood stasis in the respective kidney regions.

Blood stasis occurred in all of the four evaluated categories of vascular diameters; however, this was clearly more often after the application of Iopromide than after the application of Iodixanol (*p* < 0.001).

#### 3.1.2. Correlation Between Blood Stasis and Kidney Regions

**Figure 3:**
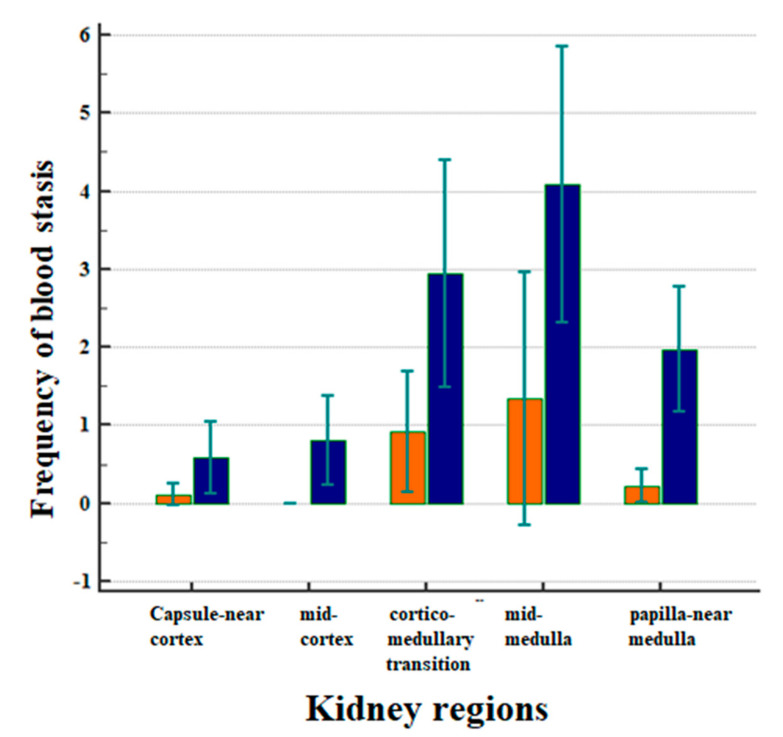
Correlation between the frequency of blood stasis and the kidney regions after CM applications in all animals, separately in the Iodixanol group (marked in orange) and for the Iopromide group (marked in blue). Arithmetic means with standard deviation are shown.

Figure 3 displays the correlation between the frequency of blood stasis and the kidney regions after CM application (Figure 3).

The frequencies of blood stasis were clearly different in the five examined kidney regions (Figure 3, *p* < 0.001). Most of the vascular obstructions occurred in the mid-medulla, followed by the cortico-medullary transition and the medulla near the papilla. The frequency of events in the medulla was significantly greater than in the capsule-near cortex (*p* = 0.0039) and in the mid-cortex (*p* = 0.0017). It was also greater than in the papilla-near medulla (Figure 3, *p* = 0.0375).

In all of the examined kidney regions, the administration of Iopromide was followed by a greater incidence of blood stasis compared to the administration of Iodixanol (Figure 3, *p* = 0.0001). There were no obstructive events in the middle cortex following the administration of Iodixanol.

### 3.2. Fibrin Staining According to Weigert

No fibrin was detected in and around the histologically assessed thrombi or in vessels with blood stasis, with the only exception being in the vicinity of the puncture channel made for the measurement of the cortical partial oxygen pressure (pO_2_), published earlier [18].

**Figure 4:**
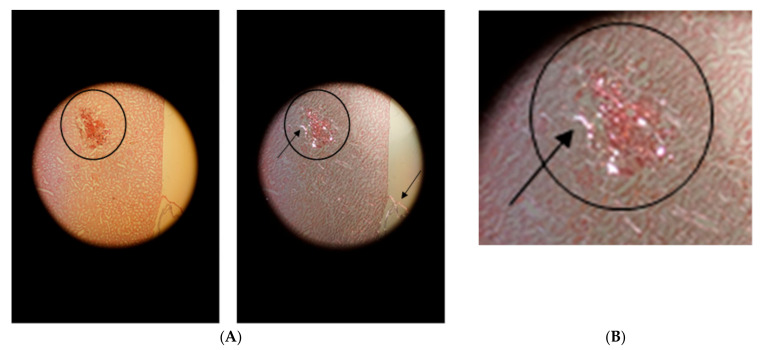
Hemorrhage in the vicinity of the puncture channel (in the circle). (**A**): on the left in transillumination and on the right in polarized light with birefringence (arrow in circle). Detection of birefringent collagen in the remnants of the kidney capsule (arrow at the kidney surface). (**B**): Primary magnification 1:100.

Figure 4A shows the hemorrhage in the vicinity of the puncture channel in the renal cortex. Fibrin was assessed by fibrin staining according to Weigert (white-grey-violet colors in transillumination) in the region marked by a circle. Birefringence (Figure 4B) was detected by applying polarized light.

**Figure 5:**
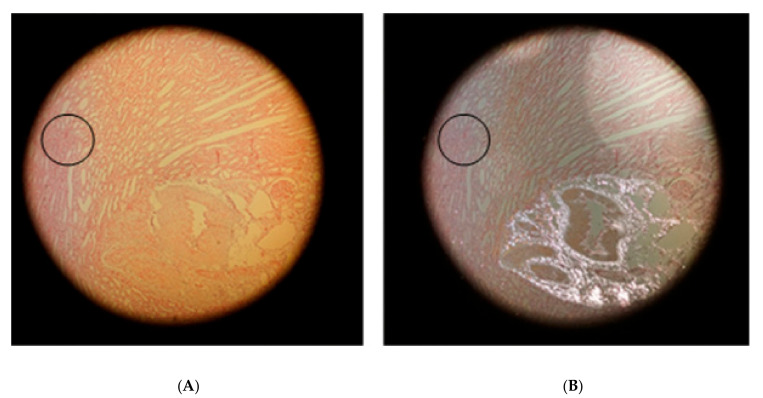
Cortico-medullary transition of a kidney without ligature of supplying vessels after CM application in transillumination (**A**) and in polarized light (**B**). Primary magnification 1:100.

Figure 5A,B show in transillumination (Figure 5A) and in polarized light (Figure 5B) the cortico-medullary transition of a kidney after CM administration. Figure 5B displays the birefringence of the fibrous collagen constituents of connective tissues accompanying vascular walls (white fiber nets), documenting the effectivity of the polarized light illumination. However, there was no birefringence in the blood-filled vessel marked by a circle, demonstrating that fibrin fibers were absent.

### 3.3. SEM Analyses

SEM analyses of 1 µm slices from explanted kidneys after in vivo application of CM displayed the effects of CM in detail.

**Figure 6:**
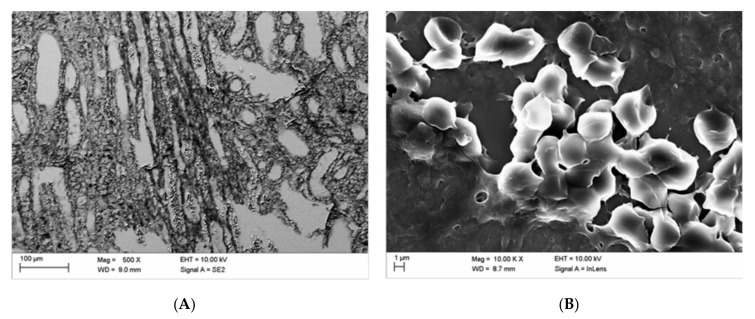
Blood congestion (**A**, middle medulla) and some echinocytically transformed (mostly in stage I according to Bessis) erythrocytes (**B**, papilla-near medulla) in kidney blood vessels after intravital application of Iodixanol.

Blood congestion in blood vessels is shown in Figure 6A. Figure 6B shows some echinocytically transformed erythrocytes with several moderate undulations according to the Bessis classification [34] stage I after administration of Iodixanol. Important to note is that fibrin was not detected in Figure 6B.

**Figure 7:**
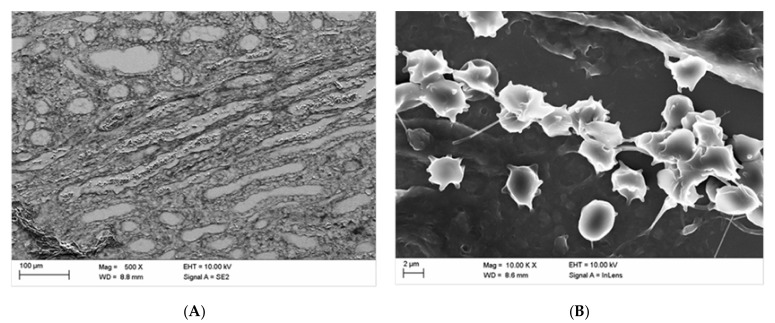
Blood congestion (**A**, middle medulla) and echinocytically transformed (mostly in stages II–III according to Bessis) erythrocytes (**B**, middle cortex) in kidney blood vessels after in vivo administration of Iopromide.

Figure 7 displays blood congestion (Figure 7A) and a greater number of echinocytically transformed erythrocytes mostly with multiple sharp spicules over its surface according to the Bessis Classification [34], stage II–III (Figure 7B) in kidney blood vessels after intravital administration of Iopromide. It is important to note is that fibrin was not detected here as well (Figure 7B).

**Figure 8:**
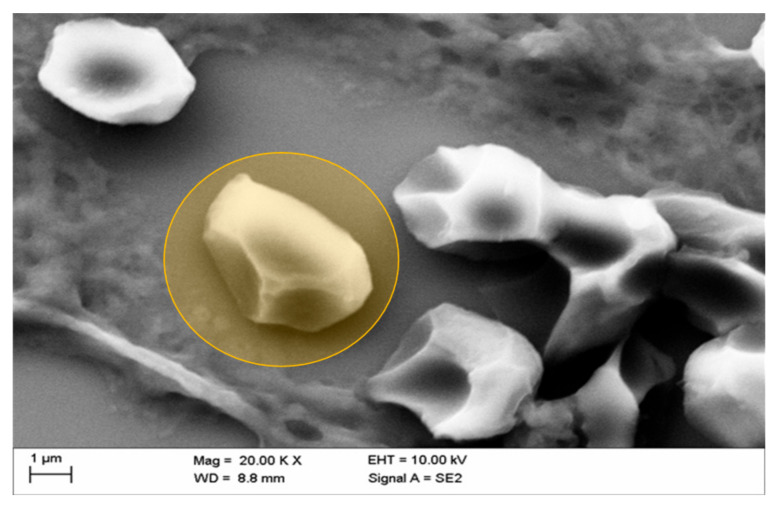
Representative picture of box-like echinocytic transformation (typically deformed erythrocyte marked in orange) of erythrocytes in kidney vessels of the middle medulla after in vivo administration of Iopromide.

Figure 8 reveals the deformation of erythrocytes in a kidney vessel, a phenomenon that has been shown before in erythrocytes in vitro after exposure of erythrocytes to a mixture of Iopromide with autologous plasma. Such deformations were found in all samples analyzed. The demonstrated intravascular “box-like” deformation was shown to be due to a considerable alteration in the submembranous cytoskeleton of erythrocytes. Furthermore, no fibrin fibers were detected here (Figure 8).

**Figure 9:**
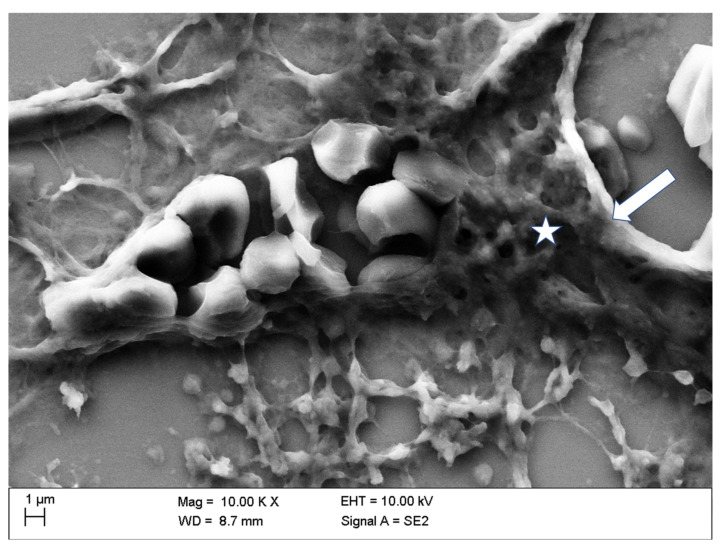
Vascular wall discontinuity (see arrow) of a kidney blood vessel in the middle medulla sealed with cascades of adhering platelets (see asterisk) in a mesh of thicker well-organized fibrin fibers (platelet thrombus), adjoining erythrocytes (intravascular), and loosely aggregated platelets with scarce fibrin constituents in the surrounding area (extravascular) after in vivo administration of Iopromide.

Figure 9 shows a discontinuity in the vascular wall of a kidney vessel in the mid-medulla region after in vivo administration of Iopromide. This serious damage of the vascular wall was sealed with cascades of adhering platelets embedded in a mesh of thin fibrin fibers and adjoining erythrocytes. Such thrombi are known to occur in vivo and not post-mortem [35] accompanied by loose platelet aggregate in the extravascular vicinity connected via thin fibrin fibers.

**Figure 10:**
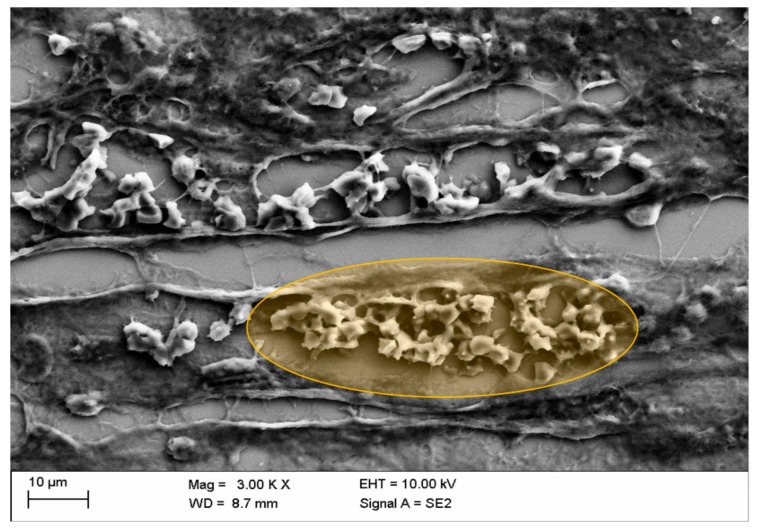
Congestion of erythrocytes in kidney blood vessels with mural aggregates of activated platelets after in vivo administration of Iopromide (○: the detail displayed in orange marks blood stasis and mural thrombus in the middle cortex).

Mural aggregates of activated platelets with erythrocytes were rarely detected in blood vessels and only after administration of Iopromide (Figure 10). This might suggest that not all of the observed effects were completely reversible.

It is important to note that the glomerular capillaries in the great majority were not affected by blood stasis for both CM.

## 4. Discussion

The histological study presented here was performed on kidneys obtained from an in vivo study on young pigs [17] with presumably healthy vasculature and undamaged kidneys. The initial serum creatinine values were low and did not increase after repeated administration of iodinated contrast media (CM) until the end of the experimental procedure (about 2 h). The protocol of CM administration along with the hydration protocol and the careful and skilled handling of the animals before and during the examination were adopted from the clinical routine in cardiac catheterization laboratories [17].

The question was whether it would be possible to unravel the “starting conditions” of CI-AKI in a large animal model in vivo, and to possibly unmask pathophysiological and pathoanatomical alterations accompanying the initial steps in the development of CI-AKI.

Pre-damage of the kidneys is thought to play a key role in CI-AKI development, but the critical factors causing kidney pre-damage and the exact mechanisms involved remain unclear.

The pathophysiological findings from the previous in vivo study demonstrated in young healthy pigs that the administration of CM can lead to an increase in the RRI [15] and to a transient disorder of the renal microcirculation as well [17]. This was dependent on the type of CM administered and was clearly more pronounced after administration of Iopromide vs. Iodixanol.

The present study demonstrated a particularly important observation, namely that repeated intra-aortal administration of CM can be associated with blood stasis within the kidney vasculature. The histological evaluation of HE-stained samples revealed that the degree of stasis was dependent on the respective kidney regions (capsule-near cortex, mid-cortex, cortico-medullary transition, mid-medulla, and papilla-near medulla). By far, the most severe changes were seen in the mid-medulla and in the cortico-medullary transition zone (see Figure 3).

Another parameter influencing the occurrence of stasis was the vascular diameter, with the highest incidence of stasis seen in blood vessels with diameters between 15 and 30 µm, followed by vessels with diameters between 30 and 300 µm and capillaries (*p* < 0.001) (see Table 2). Of note, blood stasis did not only occur in vasa recta (blood vessels with diameters between 15 and 30 µm, and 30 and 300 µm) but also in capillaries (see Table 2). This is noteworthy, in light of the previous assumption that the increase in resistance to blood flow in renal arteries depended only on the vasoconstriction in the medullary vasa recta [23].

It seems inappropriate, however, to only consider the vasa recta, since a pronounced microcirculatory disorder in different regions of the kidney after CM administration was observed using CEUS technology in a previously published investigation [17]. This study demonstrated a clear reduction of blood velocities in all paths of kidney perfusion (cortical, cortico-medullary, and medullary paths) after the intra-arterial administration of Iopromide, which became significant after the 10th CM bolus.

As demonstrated in Figure 3 and Table 2, a significant difference in the occurrence of blood stasis was assessed following intra-aortal administration of Iopromide.

The SEM analysis revealed instructive details. For the assessment of the quality of the SEM images, it should be kept in mind that the kidneys remained after explantation in dry conditions up to 30 min for X-ray examinations and were then immersion fixated (formalin) for histological preparation (HE, Weigert). The findings in the HE samples then directed the following preparation of tissue blocks for SEM examinations so that the SEM examination was findings oriented and not at random. These are not optimal conditions for the conservation of kidney tissues for SEM examination but allow an assessment of blood cells and vessel wall continuity. Artefacts due to delayed fixation after 30 min cannot be excluded. However, echinocytes and box-like deformations were solely observed after Iopromid but not after Iodixanol application. So, these cellular deteriorations are unlikely caused by the delayed fixation of the kidney. Figure 6 and Figure 7 display the echinocytic transformation of erythrocytes following intravital administration of CM. Transformation of erythrocytes has been shown to occur also in vitro when erythrocytes were exposed to CM [26,30]. The transformation induced a considerable stiffening of erythrocytes [36,37,38], thus hindering their passage in the microcirculation [19,21]. Interestingly, the echinocytic transformation of erythrocytes found in this in vivo study was significantly more pronounced following administration of Iopromide (echinocyte formation stage II–III according to Bessis [34]) than Iodixanol (echinocyte formation stage I according to Bessis [34]). Even the “box-like” transformation of erythrocytes, which so far has been identified only in vitro after their incubation in a mixture of autologous plasma and Iopromide [7], could now be clearly demonstrated intravascularly in vivo (Figure 8). Inter-endothelial discontinuity, which has previously been described in endothelial cell cultures after adding Iopromide to the cell culture medium [31,39,40,41,42], now could be detected in kidney vessels after administration of Iopromide.

The blood stasis documented in the HE-stained renal tissue samples in this investigation could be observed in greater details following the SEM examination. However, platelet aggregates were not regularly found. It must be pointed out that for this examination, young animals with apparently healthy vasculature were chosen and that these were well hydrated throughout the entire examination (as required by the ESUR guidelines 2018 [43]).

It is assumed that in patients with pre-damaged kidneys and restricted kidney function, vascular wall alterations accompanied by thrombo-aggregation could be the reason described for the rarely occurring need for dialysis following coronary angiography [44]. Due to the high reversibility of CI-AKI, advanced activation of the plasmatic coagulation was not expected. Indeed, this was not detected in the Weigert-stained samples. However, the SEM examination, as a high-resolution method, revealed a slight activation of the plasmatic coagulation in some kidney samples (Figure 9 and Figure 10).

Therefore, the fibrin-stained kidney tissues underscore our expectation. Only very few fibrin fibers were found in the Weigert fibrin-stained kidneys, with the exemption of the region of the puncture channel of the pO_2_ catheter. Scarce fibrin fibers (Figure 9) and few mural thrombi (Figure 10) were only found in Iopromide-exposed kidney vessels in SEM analyses. The absence of a noticeable activation of the plasmatic coagulation, especially the scarcity of fibrin fiber formation, is probably one of the key reasons why perfusion seems to return to normal within a short period after CM administration. This finding has been demonstrated both experimentally and in clinical practice [16,19,21,22,45,46,47,48].

It is important to note that the glomerular capillaries in the great majority were not affected by blood stasis for both CM. This could be another reason why the majority of patients after coronary angiography develop a transient deterioration of kidney function only [44].

## 5. Conclusions

According to previous in vitro studies, pathological erythrocyte deformations, such as echinocyte and box-like formation of erythrocytes, were observed also in vivo. In addition, it must be noted that blood stasis and/or thrombi could be detected in histological samples from explanted kidneys from young pigs after repeated in vivo administration of CM. Fibrin-stained samples revealed that there was no manifestation of fibrin fibers in accumulated blood or in thrombi. However, the SEM analysis revealed, in only a few cases, mural platelet aggregates within minimal fibrin meshes, only after the application of Iopromide.

## Figures and Tables

**Table 1:** Iodine concentration, osmolality, and viscosity of the respective contrast media.

Contrast Media	Iodine Concentration [mg/mL]	Osmolality [mOsmol/kg Water]	Viscosity (37 °C) [mPa.s]
Iodixanol (Visipaque™)	320	290	11.4
Iopromide (Ultravist™)	370	770	9.5

**Table 2:** Frequencies of blood stasis in all measured kidney regions and specified for the described categories of vascular diameters.

Renal Region	Capillaries	Capillaries	Vessel Ø 15–30 µm	Vessel Ø 15–30 µm	Vessel Ø 30–300 µm	Vessel Ø 30–300 µm	Vessel Ø >300 µm	Vessel Ø >300 µm
	Iodixanol	Iopromide	Iodixanol	Iopromide	Iodixanol	Iopromide	Iodixanol	Iopromide
**Capsule-Near Cortex**	0	10	4	2	0	8	0	1
**Mid-Cortex**	0	5	0	11	0	10	0	3
**Cortico-Medullary Transition**	0	20	27	46	2	44	0	0
**Mid-Medulla**	2	20	18	95	1	59	0	0
**Papilla-Near Medulla**	1	11	7	35	0	25	0	0
